# TLR4 Inhibits Mesenchymal Stem Cell (MSC) STAT3 Activation and Thereby Exerts Deleterious Effects on MSC–Mediated Cardioprotection

**DOI:** 10.1371/journal.pone.0014206

**Published:** 2010-12-03

**Authors:** Yue Wang, Aaron M. Abarbanell, Jeremy L. Herrmann, Brent R. Weil, Mariuxi C. Manukyan, Jeffrey A. Poynter, Daniel R. Meldrum

**Affiliations:** 1 Department of Surgery, Indiana University School of Medicine, Indianapolis, Indiana, United States of America; 2 Department of Cellular and Integrative Physiology, Indiana University School of Medicine, Indianapolis, Indiana, United States of America; 3 Center for Immunobiology, Indiana University School of Medicine, Indianapolis, Indiana, United States of America; City of Hope National Medical Center, United States of America

## Abstract

**Background:**

Bone marrow-derived mesenchymal stem cells (MSC) improve myocardial recovery after ischemia/reperfusion (I/R) injury. These effects are mediated in part by the paracrine secretion of angiogenic and tissue growth-promoting factors. Toll-like receptor 4 (TLR4) is expressed by MSC and induces apoptosis and inhibits proliferation in neuronal progenitors as well as many other cell types. It is unknown whether knock-out (KO) of TLR4 will change the paracrine properties of MSC and in turn improve MSC-associated myocardial protection.

**Methodology/Principal Findings:**

This study explored the effect of MSC TLR4 on the secretion of angiogenic factors and chemokines *in vitro* by using ELISA and cytokine array assays and investigated the role of TLR4 on MSC-mediated myocardial recovery after I/R injury in an isolated rat heart model. We observed that MSC isolated from TLR4 KO mice exhibited a greater degree of cardioprotection in a rat model of myocardial I/R injury. This enhanced protection was associated with increased angiogenic factor production, proliferation and differentiation. TLR4-dificiency was also associated with decreased phosphorylation of PI-3K and AKT, but increased activation of STAT3. siRNA targeting of STAT3 resulted in attenuation of the enhanced cardioprotection of TLR4-deficient MSC.

**Conclusions/Significance:**

This study indicates that TLR4 exerts deleterious effects on MSC-derived cardioprotection following I/R by a STAT3 inhibitory mechanism.

## Introduction

Accumulating evidence indicates that MSC mediate their beneficial effects at least in part through the production of cytoprotective paracrine factors that reduce inflammation, decrease apoptosis and improve myocardial function [1], [2], [3], [4], [5], [6], [7], [8], [9]. These paracrine factors also play a pivotal role in MSC-mediated chemotaxis and angiogenesis [10], [11], [12], [13], [14]. Vascular endothelial growth factor (VEGF), hepatocyte growth factor (HGF) and insulin-like growth factor-1 (IGF-1) all have been implicated in MSC-mediated functional recovery of the heart after ischemic injury [5], [15], [16], [17]. However, in spite of these encouraging experimental results, clinical trials have demonstrated that these stem cells have a modest and short-lived benefit in restoring cardiac function following ischemia [18], [19], [20], [21], [22]. Therefore, there is a critical need to discern the mechanisms by which MSC mediate their experimental benefits, and the ways in which their function can be optimized for potential clinical benefit.

The inflammatory environment of the injured heart likely restricts the therapeutic benefit of the implanted MSC. Thus, genetic modification of stem cells may represent an important strategy in improving stem cell therapy. Toll like receptors 4 (TLR4) are expressed by MSC and may play an deleterious role in MSC-mediated protection [23]. TLR4 is known to induce apoptosis and inhibit proliferation in neuronal progenitors as well as many other cell types [24], [25]. Conversely, the deficiency of TLR4 or its downstream effectors results in decreased proinflammatory cytokine production and increased differentiation and proliferation [23], [25]. Although it is well established that MSC exert a protective role in hearts following acute ischemia and reperfusion (I/R) injury [3], [5], [15], [17], [26], it is unknown whether knock-out of TLR4 will increase MSC-mediated cardioprotection. Furthermore, it is unclear if TLR4 regulates the paracrine properties of MSC. Finally, it is not known whether TLR4 regulates the activation of intracellular kinases such as STAT3, MAPK, PI-3K and AKT, whose activity has been shown to modulate the release of cytoprotective paracrine factors from MSC [16], [27], [28]. We therefore hypothesized that TLR4-knockout (TLR4KO) MSC would confer a greater degree of cardioprotection against acute I/R by increasing angiogenic and tissue growth-promoting factor secretion, and that the protective effects of TLR4KO MSC would be associated with differential activation of the PI-3K, AKT, MAPK or STAT3 signaling pathways.

## Results

### MSC isolated from WT or TLR4KO mice express Sca-1, CD44 and are capable of differentiating into adipocytes and osteocytes

**Figure 1:**
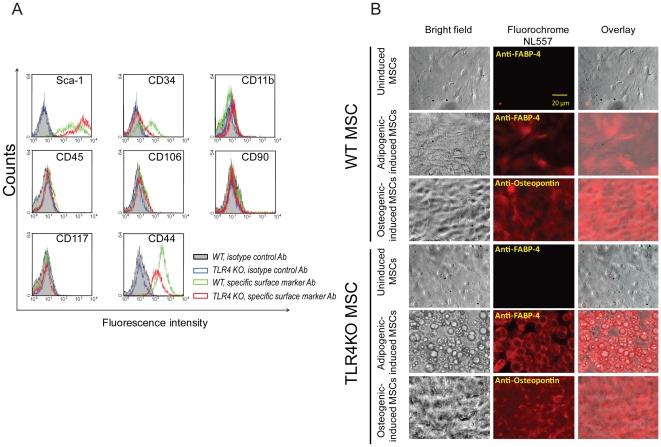
MSC express Sca-1 and CD44 and are capable of differentiating into adipocytes and osteocytes. A: WT and TLR4KO MSC were incubated with specific surface marker antibodies or isotype control antibodies and subjected to flow cytometry analysis. B: MSC in both genotypes were cultured with induction media for 3–4 weeks to induce cell differentiation. The expression of FABP-4 (adipocyte-marker) or osteopontin (osteocyte-marker) was examined using a florescence microscopy (NL557-positive red staining). Representative fields are shown at 100× magnification.

In order to demonstrate that the cells used in this study were indeed MSC, we determined whether the cells isolated from the mouse bone marrow expressed MSC surface markers and whether they could be induced to differentiate. Based on our flow cytometry data, the cells were negative for CD45, CD11b, CD106, CD117, CD90 and positive for stem cell antigen-1 (Sca-1) and the mesenchymal stem cell marker CD44 (Fig. 1A). Interestingly, a small population of MSC in both genotypes was positive for CD34 (Fig. 1A). To assess their ability to differentiate into mesodermal lineages, MSC were cultured with media containing adipogenic or osteogenic supplements. As shown in Fig. 1B, MSC of both genotypes cultured without differentiation media (uninduced) did not express fatty acid binding protein 4 (FABP-4, a marker specific to adipocytes) or osteopontin (specific to osteocytes). In contrast, cells grown in media supplemented with adipogenic or osteogenic induction agents did express FABP-4 or osteopontin. Compared with wildtype (WT) MSC, more TLR4KO MSC had differentiated into adipocytes and osteocytes when assessed under the microscope (Fig. 1B). Thus these isolated stromal cells possessed the phenotypic cell surface markers of MSC and were able to differentiate into adipocytes and osteoblasts, indicating that these cells are MSC.

### TLR4KO MSC have a higher rate of proliferation than WT MSC

**Figure 2:**
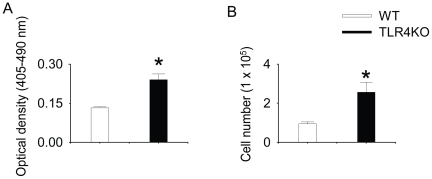
TLR4KO MSC have a higher rate of proliferation than WT MSC. A: WT and TLR4KO MSC were plated in 96-well plate (5000 cells per well) for 24 h and then incubated with BrdU (10 µM) for an additional 24 h. BrdU incorporation was quantified after incubation. N = 5–6/group. B: WT and TLR4KO MSC were plated in 12-well plates (0.5×10^5^ cell/well). After 24 h, cells were trypsinized and counted using an automatic cell counter. Data are the combination of five independent experiments. Results are mean ± SEM, * *p*<0.05 vs. WT control.

To investigate whether TLR4KO MSC exhibited altered rate of proliferation, the proliferation rates of both genotypes were examined by 1) BrdU incorporation and 2) direct cell counting. We observed that TLR4KO MSC incorporated two-fold more BrdU as compared to WT MSC (Figure 2A). Corroborating this result, the numbers of TLR4KO MSC were at least two-fold greater when compared with WT MSC cultured under similar condition, indicating increased proliferation in the TLR4KO cells (Figure 2B).

### The production of VEGF, HGF and IGF-1 in WT and TLR4KO MSC

**Figure 3:**
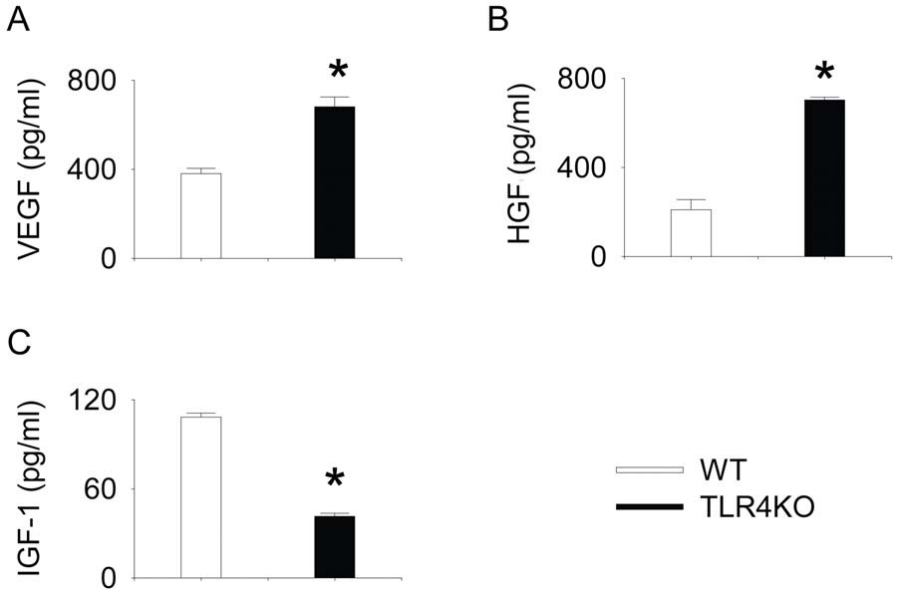
TLR4KO MSC produced higher levels of VEGF and HGF but lower levels of IGF-1 compared with WT MSC. WT and TLR4KO MSC were plated in 12-well plates (0.5×10^5^ cell/well). After 24 h, supernatant was collected and the total amounts of VEGF (A), HGF (B) and IGF-1(C) were measured. Data are representative of at least three independent experiments. Results are mean ± SEM; n = 3/group. * *p*<0.05 vs. WT control.

0.5×10^5^ cells·well^−1^·ml^−1^ from WT or TLR4KO MSC were plated and the supernatants collected after 24 h incubation and measured for VEGF, HGF and IGF-1 production. TLR4KO MSC produced significantly more total VEGF compared to WT MSC over a 24 h period (Fig. 3A). TLR4KO MSC also produced significantly more total HGF (Fig. 3B). In contrast, IGF-1 production was significantly lower in TLR4KO MSC (Fig. 3C). Immediately after supernatant collection, the cells were trypsinized and counted. The number of cells per well was (0.84±0.05) ×10^5^ and (1.5±0.11) ×10^5^ for WT and TLR4KO MSC, respectively (n = 3).

### The differential chemokine expression in WT and TLR4KO MSC

**Figure 4:**
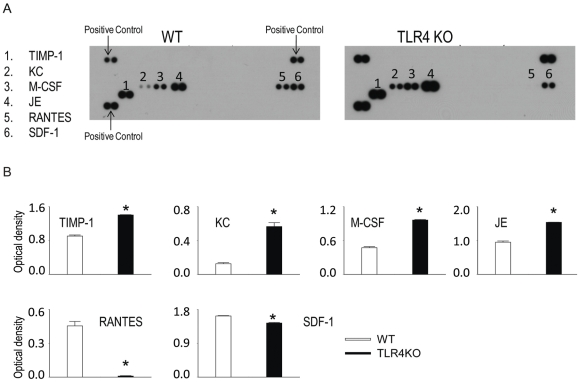
WT and TLR4KO MSC secrete various chemokines. WT and TLR4KO MSC were plated in 12-well plates (0.5×10^5^ cell/well). After 24 h, supernatants were collected for cytokine array assay. A: representative cytokine array blots are shown. B: Array signals from images of scanned X-ray films were analyzed and quantified as pixel densities. Data are the combination of two independent experiments. Results are mean ± SEM, n = 4/group, * *p*<0.05 vs. WT control.

To study the role of TLR4 in the chemokine production in MSC, the amount of chemokines in the cell supernatant was measured. 0.5×10^5^ cells·well^−1^·ml^−1^ from WT or TLR4KO MSC were plated and the supernatants collected after 24 h incubation. As shown in Fig. 4A, MSC of both genotypes were a potent source of a variety of chemokines namely TIMP metallopeptidase inhibitor 1 (TIMP-1), chemokine (C-X-C motif) ligand 1 (CXCL1/KC), macrophage colony-stimulating factor (M-CSF), mouse monocyte chemoattractant protein-1 (MCP-1/JE), the chemokine CC chemokine ligand (CCL) 5/RANTES and stromal derived factor-1 (SDF-1). The production of TIMP-1, KC, M-CSF and JE was significantly higher in TLR4KO MSC; whereas the production of RANTES and SDF-1 was lower (Fig. 4B). Immediately after supernatant collection, the cells were trypsinized and counted. The number of cells per well was (0.86±0.06) ×10^5^ and (1.9±0.2) ×10^5^ for WT and TLR4KO MSC, respectively (n = 4).

### The activation of intracellular kinases in both genotypes

**Figure 5:**
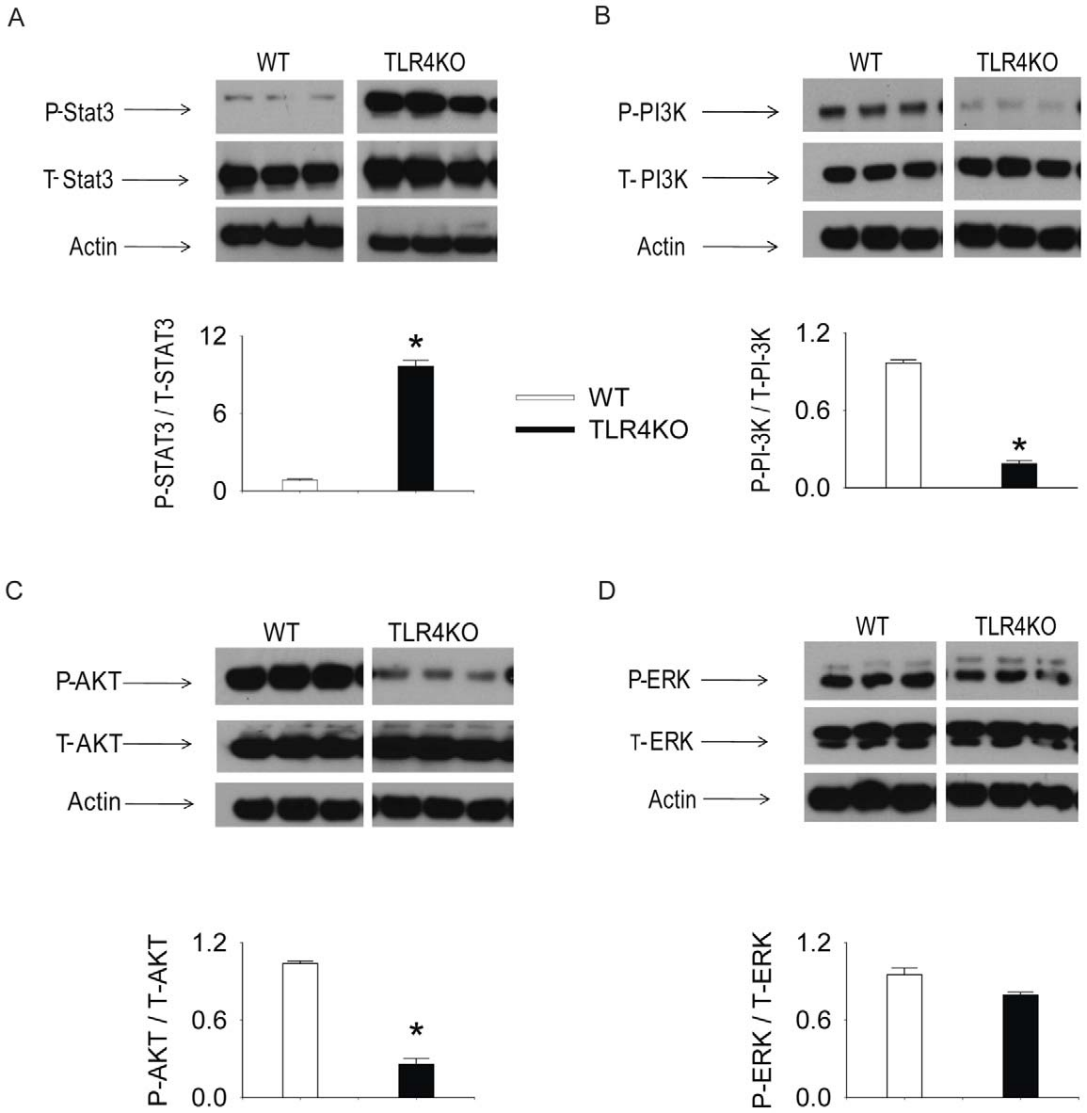
Intracellular kinase activation in WT and TLR4KO MSC. WT and TLR4KO MSC were plated in 12-well plates (0.05×10^6^ cell/well). After 24 h, cell lysates were collected and the activation of of STAT3 (A), PI-3K (B), AKT (C) and ERK (D) were measured by Western blot. The upper panels in each subfigure show the representative Western blots. The lower panels of each subfigure represent the quantified signal ratio of the activated form to total form (or actin loading control) from the Western blot. The data represent three independent experiments. Results are mean ± SEM, n = 3–4 group, * *p*<0.05 vs. WT control. Representative blot out of 4 independent experiments are shown.

We reported previously that the activity of intracellular kinases, such as PI-3K-AKT, MAPK and STAT3 modulate the paracrine properties of MSC [16], [27], [28]. Therefore, in this study, the activity of these signaling molecules was examined in both genotypes. As shown in Fig. 5A, the phosphorylated STAT3 was significantly increased in TLR4KO MSC. In contrast, TLR4KO MSC have decreased activation of the PI-3K-AKT signaling pathway (Fig. 5B–C). TLR4 deficiency did not change ERK activation (Fig. 5D).

### Knockout of TLR4 improves MSC-mediated cardioprotection following ischemic injury

**Figure 6:**
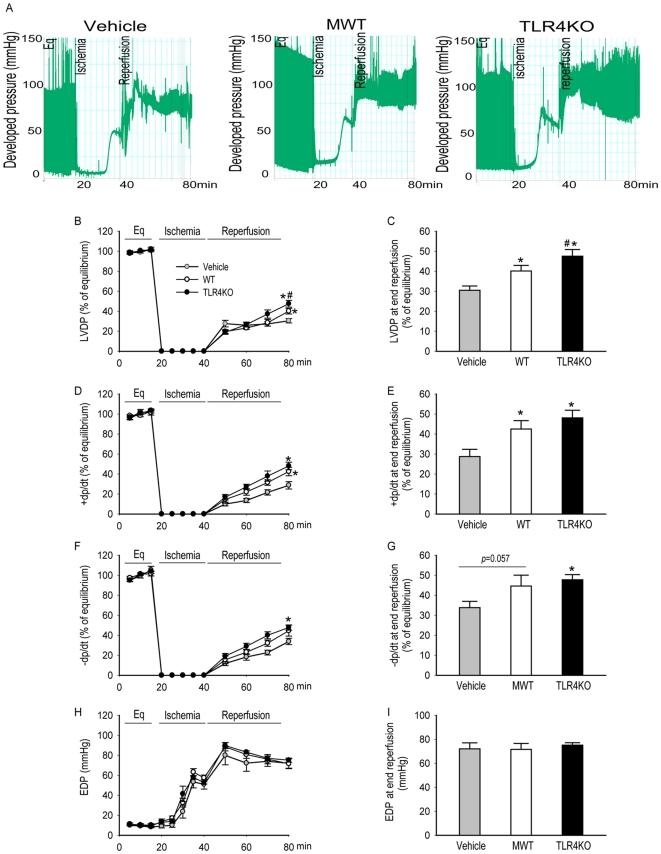
Intracoronary infusion of TLR4KO MSC provided greater cardioprotection compared to WT MSC. One million WT or TLR4KO MSC were infused into the coronary circulation prior to global ischemia. The left ventricular function in vehicle (grey, n = 8 rat hearts), WT MSC (white, n = 6), and TLR4KO MSC treated hearts (black, n = 6) is presented. A: representative recording trace from the hearts infused with vehicle, MWT or TLR4KO MSC. The line-graphs represent left ventricular function parameters over time including LVDP (% of equilibrium, B), + dP/dt (% of equilibrium, D), − dP/dt (% of equilibrium, F) and EDP (H). Recovery at end reperfusion is indicated in bar graph form: LVDP (C), + dP/dt (E), −dP/dt (G) and EDP (I). All results are reported as the mean ± SEM. * *p*<0.05 vs. Vehicle; # *p*<0.05 vs. WT MSC group.

In order to examine whether TLR4 deficiency improved MSC-mediated cardioprotection in an isolated rat heart I/R model, 1 million MSC of either genotype was infused into the coronary system before ischemic injury. The indexes of left ventricular performance, namely, left ventricular developed pressure (LVDP), end diastolic pressure (EDP, indicating the compliance of ventricular muscle), maximum +dP/dt (rate of pressure rise during ventricular contraction) and −dP/dt (rate of pressure fall during ventricular relaxation) were monitored constantly. Figure 6A is the representative recording trace of developed pressure of isolated rat hearts of different groups. Treatment of hearts with MSC of either genotype prior to ischemia resulted in significantly improved post-ischemic functional recovery as compared to vehicle controls. This was demonstrated, specifically, via improved LVDP, +dp/dt and −dp/dt in the experimental groups as compared to the vehicle controls (Figure 6B–I). Compared to WT MSC, TLR4KO MSC conferred a greater degree of myocardial protection following I/R. The hearts infused with TLR4KO MSC had higher LVDP compared with those infused with WT MSC after I/R (Figure 6A–F). However, the EDP was not different among all tested groups (Figure 6G–H). The similar value of EDP of hearts in different groups may suggest the equivalence of injury after I/R.

### siRNA specifically targeting STAT3 decreased STAT3 expression in TLR4 KO MSC

**Figure 7:**
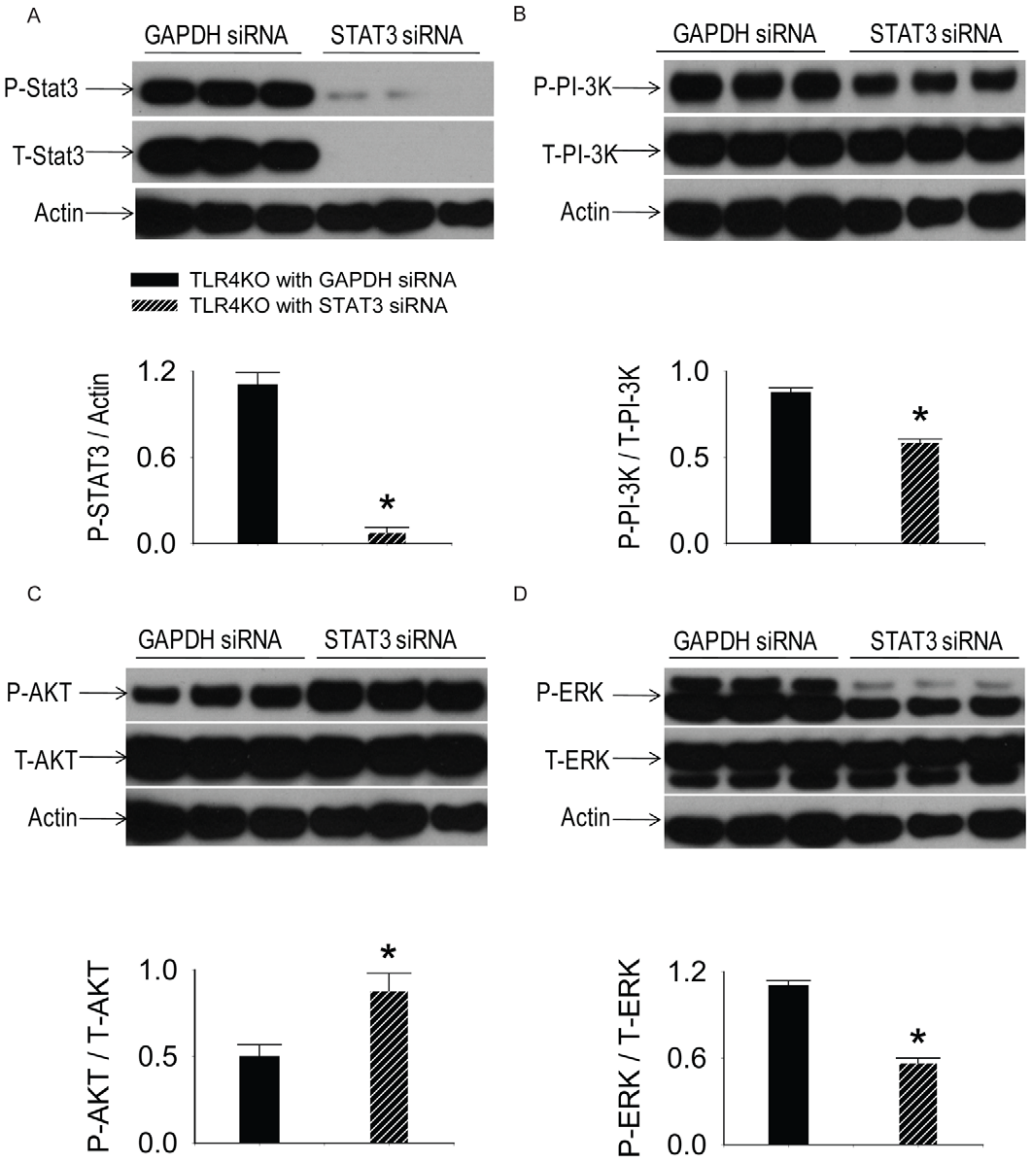
The role of STAT3 siRNA on the activation of STAT3, PI-3K-AKT and MAPK pathway. TLR4KO MSC were plated in 12-well plates at 0.5×10^5^cells·well^−1^·ml^−1^24 h prior to siRNA transfection. The lipofectamine-siRNA complex (100 nM) was added on culture day 2. Medium was then changed after 2 days (day 4 in culture) of transfection. 3 days after transfection (day 5 in culture), cell extracts were prepared for performing Western blots. The activity of STAT3, PI-3K, AKT and ERK was examined by using Western blot. The upper panels in each subfigure show the representative Western blots. The lower panels of each subfigure demonstrate the quantified signals from images of the Western blot. A: STAT3 specific siRNA, but not GAPDH siRNA decreased phosphorylated (P) STAT3 and total (T) STAT3. B–D: STAT3 siRNA suppressed the activation of PI-3K and ERK, but increased the activation of AKT. N = 3 (representative blot out of 6 independent experiments are shown).

To investigate the role of STAT3 in the paracrine signaling and the cardioprotection of TLR4KO MSC following ischemia and reperfusion injury, STAT3 siRNA was used to suppress the expression of STAT3. Three days after transfection, the expression of STAT3 (86 KD) was decreased in groups treated with STAT3 siRNA compared to the cells transfected with GAPDH siRNA (Fig. 7A). siRNA targeting of STAT3 suppressed the activation of PI-3K and ERK, but increased the phosphorylation of AKT (Fig. 7B–D).

### siRNA targeting of STAT3 decreased the production of growth factors in TLR4KO MSC

**Figure 8:**
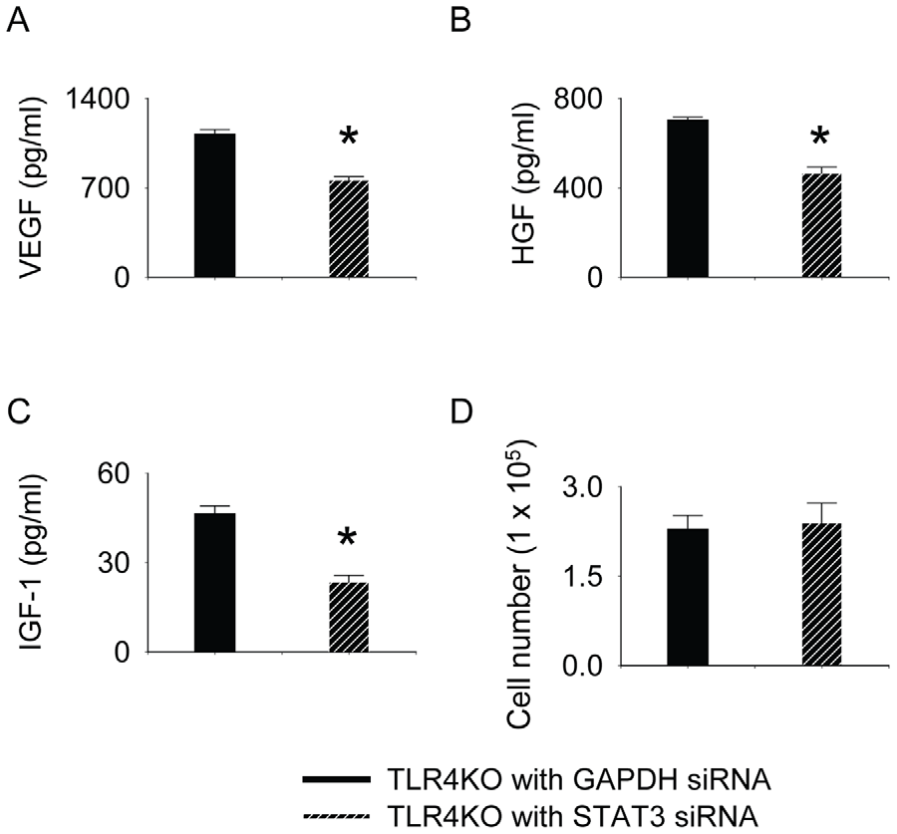
TLR4KO MSC ablated with STAT3 produced lower levels of VEGF, HGF and IGF-1 compared with TLR4KO MSC ablated with GAPDH. TLR4KO MSC were plated in 12-well plates at 0.5×10^5^cells·well^−1^·ml^−1^24 h prior to siRNA transfection. The lipofectamine-siRNA complex (100 nM) was then added on culture day 2. Medium was then changed after 2 days (day 4 in culture) of transfection. 3 days after transfection (day 5 in culture), the supernatant was collected from TLR4KO MSC either transfected with STAT3 siRNA or GAPDH siRNA and was analyzed by using VEGF, HGF and IGF-1 ELISA. Total amounts of VEGF (A), HGF (B) and IGF-1(C) were examined. siRNA targeting of STAT3 did not change the proliferation of TLR4KO cells (D). Data are representative of at least three independent experiments. Results are mean ± SEM; n = 3/group. * *p*<0.05 vs. GAPDH ablated group.

siRNA targetingof STAT3 in TLR4KO MSC decreased the production of VEGF, HGF and IGF-1. As shown in Fig. 8A–C, the release of VEGF, HGF and IGF-1 from TLR4 KO MSC transfected with STAT3 siRNA was significantly lower compared with these transfected with GAPDH siRNA. However, siRNA targeting of STAT3 did not suppress the proliferation of TLR4KO cells (Figure 8D).

### Knock-down of STAT3 altered the chemokine expression profile in TLR4KO MSC

**Figure 9:**
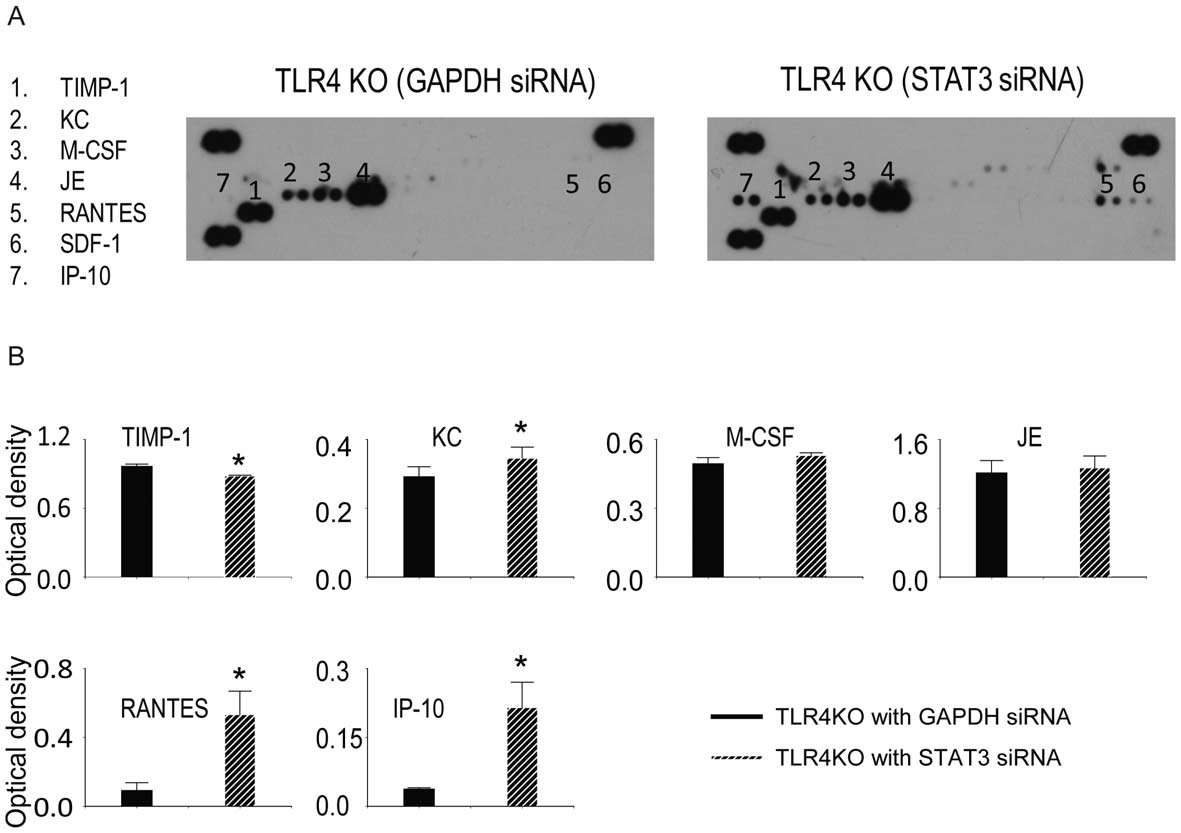
siRNA targeting of STAT3 changed the chemokine expression profile in TLR4KO MSC. TLR4KO MSC were plated in 12-well plates at 0.5×10^5^cells·well^−1^·ml^−1^24 h prior to siRNA transfection. The lipofectamine-siRNA complex (100 nM) was then added on culture day 2. Medium was then changed after 2 days (day 4 in culture) of transfection. 3 days after transfection (day 5 in culture), the supernatant was collected from TLR4KO MSC either transfected with STAT3 siRNA or GAPDH siRNA and analyzed using a cytokine array assay. A: representative cytokine array blots are shown. B: Array signals from images of scanned X-ray films were analyzed and qualified as pixel densities. Data are the combination of two independent experiments. Results are mean ± SEM, n = 4/group, * *p*<0.05 vs. GAPDH-ablated TLR4KO group.

To investigate the role of STAT3 in the cytokine production in TLR4KO MSC, the levels of cytokines in the cell supernatants were measured. siRNA targeting of STAT3 decreased TIMP-1 expression but increased the expression of KC, RANTES and Chemokine (C-X-C motif) ligand 10 (CXCL10/IP-10) (Fig. 9).

### siRNA targeting of STAT3 attenuated the cardioprotection associated withTLR4KO MSC treatment

**Figure 10:**
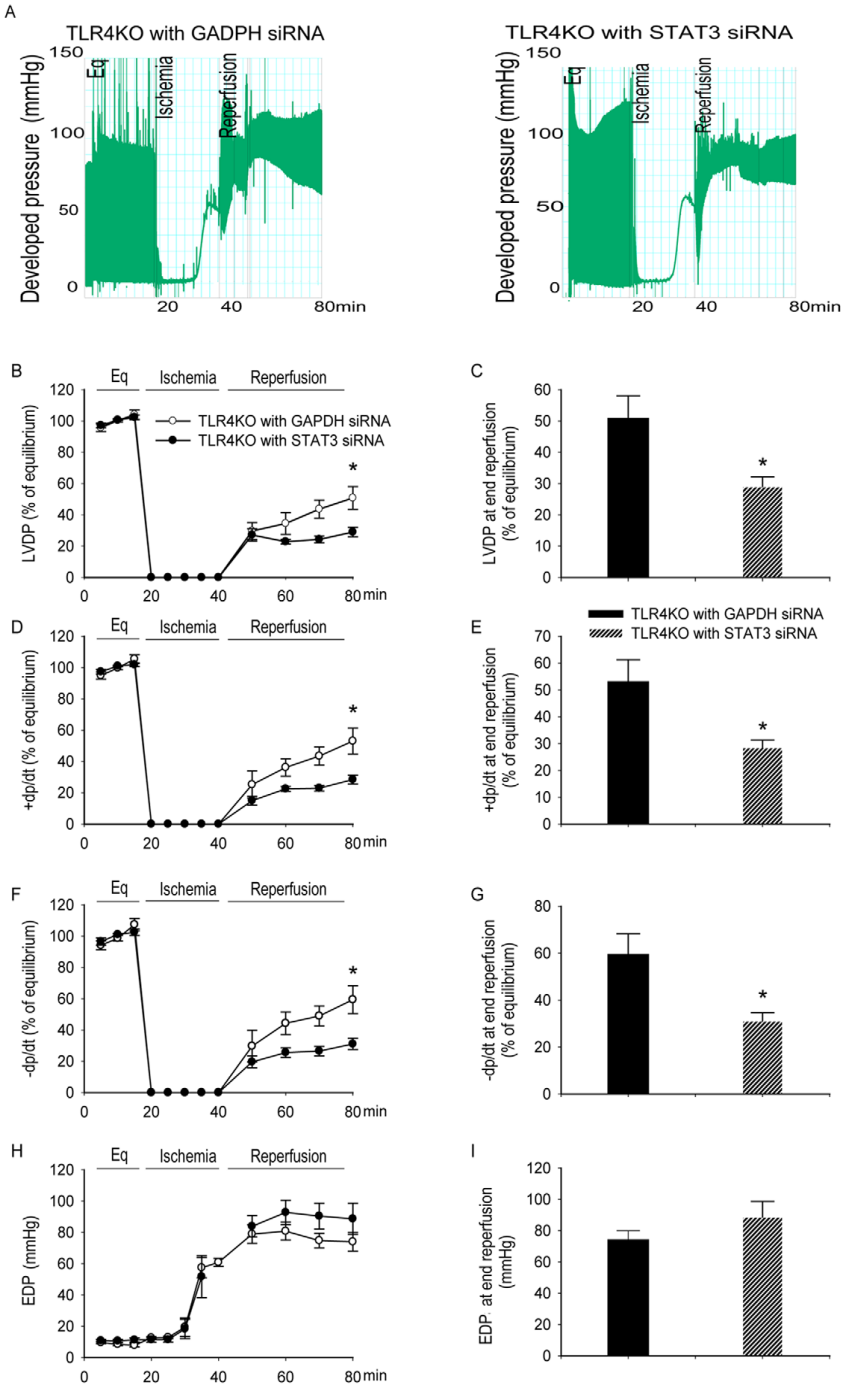
siRNA targeting of STAT3 abolished cardioprotection of TLR4KO MSC. One million TLR4KO MSC transfected with STAT3 siRNA or GAPDH siRNA were infused into the coronary circulation prior to global ischemia. Intracoronary infusion of TLR4KO MSC transfected with GAPDH siRNA provided greater cardioprotection compared to TLR4KO MSC transfected with STAT3 siRNA. The left ventricular function of hearts infused with GAPDH ablated TLR4KO MSC (black, n = 4) and STAT3 ablated TLR4KO MSC (hatched bars, n = 4) is presented. A: representative trace recorded from hearts infused with GAPDH or STAT3 ablated MSC. The line-graphs represent left ventricular function parameters over time including LVDP (% of equilibrium, B), + dP/dt (% of equilibrium, D), − dP/dt (% of equilibrium, F) and EDP (H). Recovery at end reperfusion is indicated in bar graph form: LVDP (C), + dP/dt (E), −dP/dt (G) and EDP (I). All results are reported as the mean ± SEM. * *p*<0.05 vs. GAPDH group.

Transfection of STAT3 siRNA significantly decreased the myocardial protection associated with TLR4KO MSC. Figure 10A shows the representative recording trace. The hearts infused with STAT3 ablated TLR4KO MSC exhibited less post-ischemic functional recovery as compared those infused with GAPDH ablated TLR4KO MSC. This decreased functional recovery was evidenced by decreased LVDP, +dp/dt and −dp/dt in the STAT3 siRNA groups as compared to the GAPDH groups (figure 10B–G). The EDP was not different between the tested groups (Figure 10H–I).

## Discussion

Delineating the factors and mechanisms that regulate the paracrine properties, proliferation, and differentiation of MSC is crucial as it may allow us to optimize MSC function prior to therapeutic use. While TLR4 has received much attention for its deleterious roles in I/R injury of heart and multiple other organs, such as liver [29], lung [30], kidney [31], and brain [32], accumulating evidence indicates that TLR4 may suppress the proliferation, survival, cytokine production, differentiation and immunomodulatory activity of MSC and other progenitors. For example, the deficiency of TLR4 or its downstream effectors decreases MSC proinflammatory cytokine production, increases survival, differentiation and proliferation of murine MSC [23], [25], [33]. Interestingly, murine MSC may exert their immunomodulatory effect by contact with the surrounding T-cells, whereas human MSC suppress the proliferation of host T-cells by releasing soluble factors, such as Jagged-1, transforming growth factor β and HGF [10], [34], [35]. It remains to be elucidated whether the deficiency of TLR4 improves the survival of implanted MSC in target tissue by escaping host immune system.

In this study, we demonstrated that TLR4KO MSC exhibited a greater capability to differentiate, proliferate and produce more angiogenic factors such as VEGF, HGF, chemokine KC and JE compared to their WT counterparts. These observations about TLR4KO MSC were associated with improved myocardial recovery in MSC treated heart in an isolated heart model of acute global I/R.

It is unlikely that the improved myocardial recovery observed in our study resulted from the expression of cell surface markers as both WT and TLR4KO MSC displayed similar phenotypic expression. Interestingly, a population of WT MSC demonstrated positive CD34 staining. CD34 has been widely used as a surrogate marker for hematopoietic stem cells, however, the CD34-positive cells in our preparation are unlikely to be contaminated hematopoietic stem cells due to the lack of other hematopoietic stem cell markers such as CD45 and CD11b. In addition, CD34 has been reported to be expressed on both human and murine mesenchymal stem cells [36], [37], [38]. These CD34-positive MSC exhibited a greater expression of angiogenesis-related genes, including fibroblast growth factor 7 and hepatoma-derived growth factors [36]. Whether these CD34-positive cells used in our study can lead to neovascularization in ischemic heart remains unknown and may warrant further investigation in a chronic infarction model.

Despite the common cell surface markers between WT and TLR4KO MSC, TLR4KO MSC proliferated more rapidly and could be induced to differentiate to a greater degree. These results are consistent with previous reports that the deficiency of TLR4 or its downstream adaptors, MyD88 and TICAM1 resulted in enhanced proliferation and neuronal differentiation in adult neural and retinal progenitor cells *in vivo* [25], [33]. The increased rate of proliferation may result from increased basal production of VEGF [39], HGF [40], TIMP-1 [41], KC [42], M-CSF [43], and JE [44] whose activity have been demonstrated to increase the proliferation of progenitor cells and many other cell types. Interestingly, VEGF [45], HGF [46], TIMP-1 [47], KC [48], M-CSF, [49] and JE [50] are also potent inducers of cell differentiation. These previously reported results together with our observations suggest that TLR4KO MSC may have an increased ability to proliferate and differentiate into end-organ cells via VEGF, HGF, TIMP-1, KC, M-CSF, and JE-dependent mechanisms. However, some caution must be taken as Rolls et al observed that TLR4 deficient cells after differentiation had decreased rates of survival, suggesting that other factors control the long-term fate of these differentiated cells [25]. Therefore, it is important to identify the factors that promote the survival of the differentiated MSC. Moreover, in order to optimize MSC-mediated protection, it is essential to regulate the balance between proliferation and differentiation. Excessive proliferation may lead to tumor formation whereas overly rapid differentiation may deplete the population of implanted stem cells. While our model of acute I/R answers the important question of how MSC function in the setting of global I/R as encountered during cardiac surgery or heart transplantation, however, it is not suited for studying the survival and differentiation of TLR4KO MSC implanted in ischemic heart tissue. A chronic infarct model such as the left anterior coronary artery ligation model is planned in the future investigations.

It is well established that MSC are capable of producing large quantities of cytoprotective growth factors and angiogenic chemokines that reduce inflammation, apoptosis, promote angiogenesis and improve end organ function [1], [2], [3], [4], [5]. However, the host inflammatory response and other endogenous molecules released in response to tissue injury may decrease MSC-mediated protection. Therefore, genetically modified MSC with increased production of angiogenic growth factors and anti-inflammatory cytokines as well as decreased production of pro-inflammatory cytokines may be a better choice for cell-based therapy. In this study, we observed that the basal production of angiogenic factors such as VEGF [51], HGF [52], KC [53], and JE [54] was significantly increased in TLR4KO MSC. The increased levels of these factors can facilitate angiogenesis and promote tissue repair and wound healing. Additionally, TLR4KO MSC produced less of the inflammatory chemokine RANTES. The differential cytokine/chemokine expression profile of TLR4KO MSC may result from decreased activity of PI-3K, AKT and increased STAT3 when compared to the WT MSC. The activity of these kinases regulates the release of paracrine factors from MSC [16], [55], [56]. Our *in vitro* observations therefore suggest that TLR4KO MSC may be a better choice for cell therapy of ischemic heart disease as a result of increased production of angiogenic factors and decreased production of proinflammatory cytokines/chemokines. However, some caution must be taken as MSC derived from TLR4KO mice may carry distinct phenotypes unrelated to the function of TLR4. Moreover, we cannot rule out the possibility that a distinct cell population may have been selected as using MSC isolated from different animals. Therefore, the experiments using MSC overexpressed TLR4 or using TLR4 specific siRNA are necessary to elucidate the role of TLR4 in MSC-mediated cardioprotection in future investigations.

In this study pre-ischemic intracoronary infusion of TLR4KO MSC improved post-ischemic heart function as evidenced by increased LVDP, +dp/dt and −dp/dt compared to WT MSC and the vehicle treatment groups. Since these protective effects occur less than 1 hour after injury, it is unlikely that the cardioprotective benefit is due to any meaningful amount of donor MSC differentiation and regeneration. The acute MSC-mediated protection in this study is more likely the result of paracrine factors released from these cells. These factors may exert their cytoprotective effects by binding to their respective receptors on cardiomyocytes and/or other surrounding cells.

The mechanism for the increased levels of paracrine factors appears to involve STAT3. At baseline, STAT3 levels were significantly higher in the TLR4KO MSC. siRNA targeting of STAT3 in TLR4KO MSC decreased the expression of VEGF, HGF, IGF-1 and TIMP-1, but increased levels of KC, IP-10 and the proinflammatory chemokine RANTES. In addition, siRNA targeting of STAT3 abolished the difference in functional recovery associated with TLR4KO MSC treatment, suggesting that TLR4KO MSC exert their cardioprotection by STAT3-dependent mechanisms. Overexpression of STAT3 in MSC may present a novel strategy to optimize their therapeutic potential.

## Materials and Methods

### Animals

Normal adult male Sprague-Dawley rats were obtained from Harlan (200–250 g, Indianapolis, IN). 6–8 weeks old male WT mice (C57BL/10ScSnJ) and mice with a homozygous deletion of TLR4 (C57BL/10ScN) were obtained from Jackson Laboratory (Bar Harbor, ME). Mice with a homozygous deletion for the TLR4 allele are viable, fertile, normal in size and do not display any gross physical abnormalities. The animals were fed a standard diet and acclimated in a quiet quarantine room for 1 week before the experiments.

### Ethical statement

The animal protocol was reviewed and approved by the Indiana Animal Care and Use Committee of the Indiana University (Animal protocol 3583 and 3281). All animals received humane care in compliance with the “Guide for the Care and Use of Laboratory Animals” (NIH publication No. 85-23, revised 1996). All Animals received humane care in compliance with the “Guide for the Care and Use of Laboratory Animals” (NIH publication No. 85-23, revised 1985). Isolated heart from adult rats and bone marrow from adult mice were used for experiments. The Public Health Service Animal Welfares Assurance and Animal Care Forms describe the facilities in which they are maintained and methods of care in compliance with NIH guidelines.

### Preparation of MSC

A single-step stem cell purification method was employed as previously described [26], [57]. Briefly, MSC were collected from the bilateral femurs and tibias of mice after sacrifice by removing the epiphyses and flushing the shaft with complete media—Iscove's Modified Dulbecco's Medium (IMDM; Invitrogen, Carlsbad, CA) and 10% fetal bovine serum (Invitrogen)—using a syringe with a 26G needle. Cells were disaggregated by vigorous pipetting and were passed through a 30-µm nylon mesh to remove any remaining clumps of tissue. Cells were centrifuged for 5 min at 500 g at 24°C. The cell pellet was then re-suspended and cultured in 75 cm^2^ culture flasks in complete media at 37°C with 5% CO_2_. MSC preferentially attached to the polystyrene surface and after 48 h, non-adherent cells in suspension were discarded. Fresh complete media was added and replaced every three or four days thereafter. When the cells reached 90% confluence, MSC cultures were recovered by the addition of a solution 0.25% trypsin-EDTA (Invitrogen) and passaged. Cell passage was restricted to passages 6–10 for the experiments. Cell counts were determined using an automatic cell counter (Nexcelom cellometer^TM^ Auto T4, Bioscience LLC, Lawrence, MA).

### Flow cytometry analysis

The following primary antibodies were used: anti-Sca-1-PE, anti-CD34-PE, anti-CD11b-APC, anti-CD45-FITC, anti-CD106-FITC, anti-CD90-PE, anti-CD117-APC, anti-CD44-PE and the recommended isotype control for each fluorochrome (BD Biosciences, San Diego, CA). MSC were incubated with specific primary antibodies for 1 h and analyzed by flow cytometry with a FACSCalibur flow cytometer (BD Biosciences).

### MSC differentiation

0.5×10^5^ MSC were seeded in a 2-well glass chamber at a concentration of 0.1×10^6^/ml. After reaching confluence, MSC were induced to differentiate by using a differentiation kit (R&D Systems, Minneapolis, MN). Briefly, MSC were incubated with adipogenic or osteogenic medium for 3-4 weeks with medium replacement twice a week. After the designated incubation period, cells were stained with anti-fatty-acid binding protein 4 (FABP4) for the adipogenic group and anti-osteopontin for the osteogenic group. MSC were then incubated with a Northern Lights 557 (NL557)-conjugated secondary antibody. Uninduced MSC were used as negative controls and maintained in complete media. Cell morphology and fluorescence were examined under an inverted fluorescence microscope (Nikon TE2000U, Nikon, Melville, NY) at 100× magnification. Images were digitalized with QCapture (QImaging, Surrey, BC) and transferred to Adobe Creative Suite 4 (Adobe Systems Inc., San Jose, CA).

### 5-Bromo-2′-deoxy-uridine (BrdU) incorporation

BrdU incorporation was used to measure DNA synthesis in a colorimetric immunoassay. Briefly, MSC were plated in 96-well Corning plates (Corning, NY) at 5000 cells/well and allowed to adhere overnight in complete media at 37°C with 5% CO_2_. Subsequently, the cells were incubated with 10 µM BrdU for 24 hours. The labeled cells were then fixed with ethanol. Prior to incubation with a monoclonal antibody to BrdU, DNA was partially digested with nucleases to allow the antibody to access the incorporated BrdU. The absorbance of the samples was determined with a standard microplate (ELISA) reader at 405 nm with a reference wavelength at 490 nm.

### VEGF, HGF and IGF-1 ELISA

0.5×10^5^ cells·well^−1^·ml^−1^ from each cell line were plated and the supernatants collected after 24 h incubation and measured for VEGF, HGF and IGF-1 production using a commercially available ELISA kit (R&D). All samples and standards were measured in duplicate. The absorbance of the samples was determined with a standard microplate (ELISA) reader at 450 nm with a reference wavelength at 540 nm.

### Cytokine array assay

0.1×10^6^ cells·well^−1^·ml^−1^ from each cell line were plated and the supernatants collected after 24 h incubation and measured for cytokines, chemokines and TIMP metallopeptidase inhibitor 1 (TIMP1) production using a mouse cytokine array kit (R&D). Briefly, the array membranes immobilized with capture antibodies were blocked and then incubated with 1 ml of conditioned medium for 2 h at room temperature. After washing out the unbound proteins, the membranes were then incubated with a biotin-conjugated detection antibody cocktail. Membranes were then incubated with HRP-conjugated streptavidin for 30 min at room temperature. The signals were detected by using SuperSignal West Pico stable peroxide solution (Pierce, Rockford, IL). Signal densities were compared using TotalLab software (Nonlinear USA, Inc., Durham NC).

### Protein Isolation and Western Blot Analysis

Western blot analysis was performed to measure the activation of PI-3K, AKT, ERK, and STAT3. Cell extracts were prepared by direct lysis of cells in cold RIPA buffer (Sigma, St Louis, MO) containing a protease inhibitor cocktail and phosphatase inhibitor cocktail 2 (Sigma). Lysates were then centrifuged at 12000 rpm for 10 minutes. Protein extracts (10 µg/lane) were electrophoresed on a 4–12% Bis Tris gel (Invitrogen) and transferred to a nitrocellulose membrane. The membranes were incubated in 5% dry milk for 1 h and then incubated with primary antibodies for phospho(P)-PI-3K, total(T)-PI-3K, P-AKT, T-AKT, P-ERK, T-ERK, P-STAT3 and T-STAT3 (Cell Signaling Technology, Inc. Boston, MA) followed by incubation with horseradish peroxidase-conjugated goat anti-rabbit IgG secondary antibody and detection using SuperSignal West Pico stable peroxide solution (Pierce, Rockford, IL). Actin levels were measured to ensure equal loading of protein. Band densities were compared using TotalLab software (Nonlinear USA, Inc.).

### Isolated heart preparation (Langendorff)

Rats were anesthetized (sodium pentobarbital, 60 mg/kg i.p.) and heparinized (500 U i.p.), and hearts were rapidly excised via median sternotomy and placed in a modified 4 °C Krebs–Henseleit (KH) solution (119 mM NaCl, 20.8 mM NaHCO_3_, 11 mM dextrose, 12 mM CaCl_2_ (2H_2_O), 47 mM KCl, 11.7 mM MgSO_4_(7H_2_O), and 11.8 mM KH_2_PO_4_). The aorta was cannulated and the heart was perfused under constant pressure (mean 75 mmHg) with oxygenated (95% O_2_/5% CO_2_) Krebs–Henseleit solution (37°C). A left atrial resection was performed to allow the insertion of a water-filled latex balloon through the atrium into the ventricle. The balloon was adjusted to a desired mean end diastolic pressure (EDP) of 5–10 mmHg, and the hearts were allowed to equilibrate for 15 minutes. Pacing wires were fixed to the right atrium and the left ventricle, and the hearts were paced at approximately 6 Hz, 3V, 2 ms (350 bpm) during equilibration and reperfusion to ensure a standard heart rate between groups. Immediately prior to global ischemia, the hearts were randomly assigned to one of three treatment groups: 1) vehicle (1 ml of KH), 2) one million WT MSC or 3) one million TLR4KO MSC. The treatements were administered by intracoronary infusion through the aortic cannula over one minute. To create warm global ischemia, the perfusion to the heart was stopped at the end of the assigned treatment using a three-way stopcock above the aortic root and the heart was placed in a 37°C degassed organ bath. After 25 min of warm ischemia, the hearts were reperfused for 40 minutes. The left ventricular developed pressure (LVDP), end diastolic pressure (EDP), maximum +dP/dt (rate of pressure rise during ventricular contraction) and –dP/dt (rate of pressure fall during ventricular relaxation) were continuously recorded using a PowerLab 8 preamplifier/digitizer (AD Instruments Inc., Milford, MA) and a Mini Mac computer (Apple Computer Inc., Cupertino, CA).

### siRNA transfection

*s*iRNA that specifically targeted mouse STAT3 were designed according to the software provided by Dharmacon siDESIGN center (Dharmacon Research, Lafayette, CO). SiRNA sequences (CAGCACAACCUUCGAAGAA) corresponding to residues 517–535 of the coding region of mouse STAT3 were selected. MSC from the same starting cell isolation were transfected with the aforementioned specific STAT3 siRNA at a concentration of 100 nM. MSC transfected with siRNA targeting GAPDH were used as control (Silencer GAPDH siRNA, Ambion, TX). Transfection was performed with Lipofectamine 2000 (Invitrogen, CA) per the manufacturers' instructions. Briefly, 24 h prior to siRNA transfection, cells were plated in 12-well plates at 0.5×10^5^cells·well^−1^·ml^−1^. On culture day 2, cells were washed with Optimem media (Invitrogen) and the lipofectamine-siRNA complex (100 nM) was then added. After 1 day of transfection (day 3 in culture), the lipofectamine-siRNA complex was washed out and the IMDM medium containing 10% FBS and 1% antibiotics was added to the cells and allowed to incubate for an additional 2 days. Medium was then changed after 2 days (day 4 in culture) of transfection. 3 days after transfection (day 5 in culture), the supernatant was collected for VEGF, HGF and IGF-1 ELISA as well as cytokine array analysis. Cell extracts were prepared for performing Western blots. For the isolated heart preparation, 3 days after transfection, MSCs were trypsinized, collected and counted with the aid of the automated cell counter (Nexcelom Bioscience). One million viable cells were isolated. The cells were centrifuged at 300 *g*, the media was removed, and the cells were resuspended in 1 ml of KH solution (37°C). Over the course of 1 min immediately prior to ischemia, the MSC solution was infused into the coronary circulation.

### Reagents

All standard chemicals were obtained from Sigma.

### Data analysis

All reported values are mean ± SEM. LVDP, +/−dp/dt are presented as a percentage of the baseline. Statistical differences between the control groups and those obtained under various treatment conditions were determined using a 1-way ANOVA followed by a Holm-Sidak post hoc analysis. Values of *p*<0.05 were judged to be statistically significant.
